# Evaluating Age-Friendly Health Care Approaches in Rural Primary Care Settings: A Multi-Case, Mixed-Methods Hybrid Type 2 Effectiveness-Implementation Study

**DOI:** 10.3390/mps7050081

**Published:** 2024-10-08

**Authors:** Kathleen Brasher, Rachel Winterton, Clare Wilding, Kelly Tamang

**Affiliations:** John Richards Centre for Rural Ageing Research, La Trobe Rural Health School, Wodonga 3550, Australia; r.winterton@latrobe.edu.au (R.W.); c.wilding@latrobe.edu.au (C.W.); k.tamang@latrobe.edu.au (K.T.)

**Keywords:** age-friendly health care, primary care, older adults, rural health care, Indigo 4Ms tool

## Abstract

Maintaining and improving the health and well-being of older people in rural communities through integrated care is essential to address this cohort’s frailty risk. The Indigo 4Ms Tool for health workers is a rural-specific approach to providing care that addresses the common conditions of ageing. With Australian government funding, five small rural health services are implementing the tool. This paper describes the protocol for a hybrid type 2 implementation-effectiveness study to evaluate the tool’s impact on multidisciplinary comprehensive care planning and the implementation strategies that enhance the adoption and sustainability of the tool across diverse rural health settings.

## 1. Introduction

Globally, we are witnessing a demographic shift where people are living longer. The World Health Organization (WHO) projects that one in six people will be over 60 by 2030 [1], and the United Nations predicts that one in every four people in Europe, North America, and Southeast Asia will be 65 or above by 2024 [2]. Over 22% of the world’s population, approximately 2.1 billion people, is estimated to be above 60 by 2050 [3]. This change in age demographic is prevalent in Australia, with people over 65 years representing 16% of the total population in 2020, and this is projected to rise to 23% by 2066 [2]. The trend is similar in countries like the United State (17%), the United Kingdom (19%), and Israel (12%) [4].

Regarding the geographic distribution of older people, large proportions reside in rural areas. In some regions in the USA, more than 50% of older adults live in rural areas [5], and about 17.5% of the rural population is above 65 years old [6]. In Australia, nearly 34% of older people live outside metropolitan settings [4].

The high rates of population ageing in rural areas have implications for managing the health and well-being of older people. As we age, biological changes lead to a gradual decrease in physiological reserve. This decrease is neither linear, consistent, nor closely associated with age in years [7]. The significant population burdens of disability and death in people aged over 60 arise from common age-related losses in seeing, hearing, and moving and from chronic disease [8,9]. Frailty is an extreme consequence of the ageing process, ‘characterised by a decline in functioning across multiple physiological systems, accompanied by an increased vulnerability to stressors’ [10]. It is a dynamic, potentially reversible decline in functional ability [11]. In a global systematic review and meta-analysis, the incidence of frailty and prefrailty in community-dwelling older people was estimated at 4.3% and 15%, respectively [12]. Older people with frailty are more likely to experience falls and fractures, incontinence, repeated admissions to hospital, poorer quality of life, and early death [11].

Evidence-based clinical pathways guide the current management of chronic conditions [13,14]. However, for older people with two or more chronic conditions, evidence shows that disease-specific pathways are not feasible [15,16] given that ‘every individual recommendation made by a guideline may be rational and evidence-based, but the sum of all recommendations in an individual is not’ [17]. Moreover, following a clinical pathway for a specific disease neglects common age-related declines and the potential path to frailty, both of which can be reversed, slowed, or prevented [9,11].

Consequently, contemporary approaches to health care for older people emphasise integrated, multidisciplinary frameworks [18,19]. The 4Ms Framework, an initiative of The John A. Hartford Foundation and the Institute for Healthcare Improvement (IHI), in partnership with the American Hospital Association (AHA) and the Catholic Health Association of the United States (CHA), structures and prioritises action across four inter-related core elements: what matters, medications, mobility, and mentation. Evidence suggests that using the 4Ms improves safety and quality and reduces care costs for older people [20], while integrated care requires a multidisciplinary approach with a single assessment and shared care plan [18,21,22].

Research into implementing the 4Ms has focused on urban settings with limited evidence from rural environments [23,24,25,26]. The 4Ms have predominantly been implemented in tertiary health care settings in the United States, including acute and sub-acute care, emergency departments, primary health clinics, and aged care facilities [25]. Given the higher proportion of older people residing in rural areas alongside the geographical, workforce, and access challenges in rural settings [27,28,29], there is a need to explore and understand how a 4Ms approach is operationalised outside acute, urban health care environments.

Consequently, this paper outlines the protocol for evaluating the implementation of the ‘Indigo 4Ms Tool for Primary healthcare Workers’ [30] (the Indigo 4Ms Tool) in diverse rural Australian primary care settings. The Indigo 4Ms Framework is an Australian, rurally specific adaptation of the IHI 4Ms [31]. The Indigo 4Ms Tool was developed in 2023 through a comprehensive co-design process with older people, health, education, aged care, and community stakeholders [32]. It is consistent with two of Australia’s National Safety and Quality of Health Standards: Partnering with Consumers and Comprehensive Care Planning [33].

This paper describes the protocol for an evaluation that aims to determine the Indigo 4Ms Tool’s effectiveness in improving multidisciplinary comprehensive care planning for older people in rural primary care health service settings. Specifically, the research aims to achieve the following:

1. Establish how and in what circumstances implementing the Indigo 4Ms Tool within rural primary health settings improves the capacity of multidisciplinary teams to deliver person-centred, comprehensive care plans for older people.
2. Identify the implementation strategies that enhance the adoption, effectiveness, and sustainability of the Indigo 4Ms Tool in diverse rural health settings.

## 2. Experimental Design

This evaluation is a multi-case, mixed-methods, hybrid type 2 effectiveness-implementation study. The effectiveness of the Indigo 4Ms Tool and each health service’s implementation strategies will be evaluated concurrently [34,35]. Each health service will integrate the tool into its own care planning processes and use its own quality improvement systems for implementation, given the strong evidence that meaningful improvements are best implemented through local actions grounded in the current care delivery and collaborative practices of executives, staff, and patients [36].

**Figure 1:**
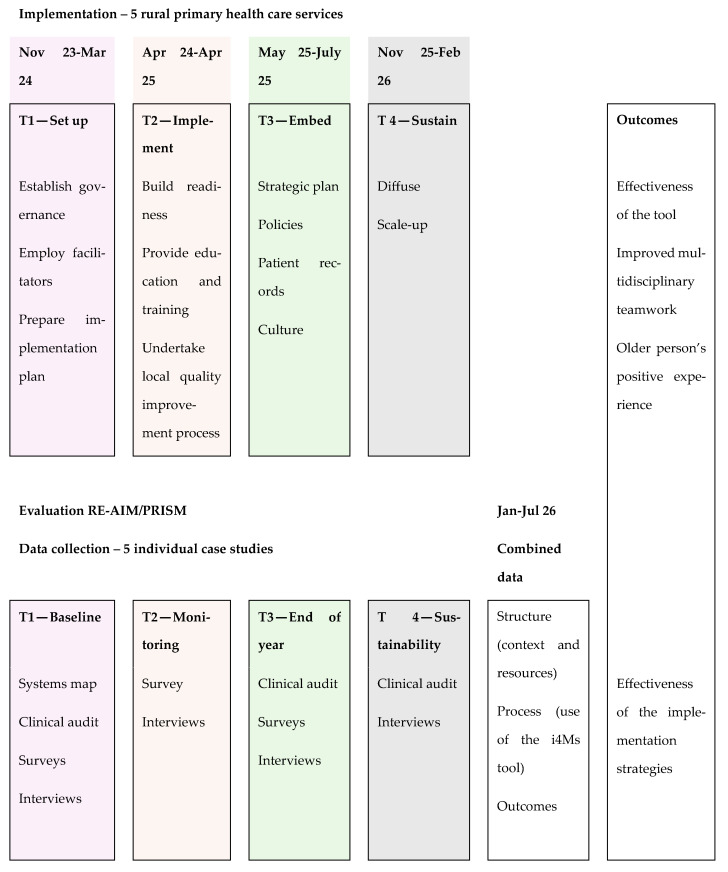
Phases of implementation and associated data collection activities.

Each health service will be studied simultaneously as an individual case [37] to investigate how the implementation of the Indigo 4Ms Tool occurs in real-world rural health care settings and what and why some approaches to using the tool or its implementation are more or less effective. Mixed methods will be used for data collection [38]. Quantitative data using surveys will precede qualitative data collection to serve the collection of qualitative data in three main ways: (1) to provide construct validity for the concepts being explored, (2) as idiographic findings to enrich data collection in interviews, and (3) to ensure concepts are used consistently across the five cases [39]. Figure 1 describes the design of the study.

### 2.1. Setting

The research will be conducted in primary health settings in five rural health services across northeast Victoria, Australia. The five rural health services have volunteered to implement the Indigo 4Ms Tool into their usual care of community-dwelling older people. Within each service, the Indigo 4Ms Tool will be implemented by multidisciplinary primary health clinicians in their routine care of community-dwelling older people in their designated health service setting (see Table 1). The nature of the primary health activities undertaken in each setting, the health workforce employed, and the demographics and health needs of older people differ across the health services. The health services reflect the breadth of primary care services across this region.

### 2.2. Analytic Framework for Evaluation

The enhanced RE-AIM/PRISM model [40] will guide the evaluation. The RE-AIM dimensions of reach (R), effectiveness (E), adoption (A), implementation (I), and maintenance (M) have been used extensively in a broad range of health settings to plan and evaluate interventions [41,42,43,44]. The Practical, Robust Implementation, and Sustainability Model (PRISM) enhances the RE-AIM framework by considering the multi-level contextual characteristics of the intervention, implementation, sustainability infrastructure, and external environment [45]. It will provide a structured approach to understanding the contexts in which the multifaceted implementation strategies were employed and what aided the adoption of the Indigo 4Ms Tool. The RE-AIM framework has been used in assessments in public health settings [46,47].

After the individual case studies have been completed, the combined findings will be used to evaluate the overall adoption of the Indigo 4Ms Tool using Donabedian’s theory (1988) of relationships between structure, process, and outcomes [48,49].

## 3. Procedure

Data will be collected at four time points over three years:

1. Initially, a baseline assessment of the comprehensive care planning currently undertaken by primary health staff and the contexts in which they work will be conducted (T1).
2. Data will be collected to monitor the use of the Indigo 4Ms Tool within the primary health implementation settings and the implementation process for one year (T2).
3. At the end of the implementation year, data will be collected to evaluate the tool’s effectiveness and implementation strategies (T3).
4. Six months after T3, data will be collected at each setting relating to the sustainability of the use of the Indigo 4Ms Tool (T4).

### 3.1. Participants

The study will engage five groups of participants from each case (study setting):

1. Health service executives (HSEs), identified by the CEO or nominee, who have strategic and operational leadership or responsibility for service delivery, primary health clinical staff, and the tool’s implementation.
2. Implementation team members (ITs) identified by the CEO or nominee as clinical governance or staff members with primary responsibility for the implementation process. Each health service will determine its implementation team’s governance, structure, membership, roles, and responsibilities.
3. Primary health clinicians (PHCs) who use the Indigo 4Ms Tool as part of their routine care for community-dwelling older people. Health services will identify the staff involved in the study. Diverse, multidisciplinary teams distinguish the rural primary health sites. These interdisciplinary teams may include physiotherapists, occupational therapists, social workers, pharmacists, home care workers, general practitioners, community nurses, diabetes educators, dietitians, mental health workers, allied health assistants, and personal trainers. The number of staff, their role in the primary health team, and their disciplines will differ across the sites.
4. Community-dwelling older people (OPs) over 55 who received routine clinical care from a member of the primary health team during the implementation period. The age range was selected to ensure that Aboriginal and Torres Strait Islander peoples will be included in the study, whether or not they self-identify. People with cognitive impairment will be supported to participate in the study in line with best practice [50]. Where relevant, researchers will seek advice from clinicians about a potential participant’s capacity to consent. Family and carers may also be involved as required or requested by the clinical staff or participant. The inclusion criteria are older people who have received primary health care services in the six months since clinicians’ use of the Indigo 4Ms Tool commenced and who require ongoing services. Exclusion criteria are people receiving palliative care or only requiring a single episode of care.
5. Facilitators (Fs) are staff members recruited and employed by each health service to support the implementation of the Indigo 4Ms Tool within the respective setting. These positions are funded through the research grant.

### 3.2. Data Collection

Quantitative and qualitative data will be collected using clinical audits, surveys, interviews, focus groups, and field notes.

#### 3.2.1. Audits

*Systems map:* The systems map will provide each organisation’s PRISM multi-level contextual data. Researchers will use the systems map audit tool (A1) to collect publicly available health services data based on the six components of a health system: service delivery, health workforce, information, medical technologies, financing, and leadership/governance [51]. These data will contextually map each health service and the primary health setting, focusing on the care of older people. CEOs will be emailed a copy of the completed systems map for veracity.

*Clinical audits:* The clinical audit tool (A2) will collect health service primary health care data relevant to the comprehensive care planning of older people. Clinical audits will be undertaken at baseline (T1), at the end of the first year of implementation (T3), and six months after T3 (T4). A research team member will attend each site to access de-identified paper or electronic health records following the protocols of the relevant health service. The clinical audit structure is based on the National Safety and Quality Health Service (NSQHS) audit tool for Comprehensive Care Standard 5 [52], with additional items relevant to identifying the discipline of staff and the bundle of care pertinent to the Indigo 4Ms [53]. Health records of eligible people over 55 who received primary care services in the two weeks before implementation, two weeks at the end of the first year of implementation, and two weeks six months later will be accessed. Data will be collected using the Australian-hosted REDcap platform.

#### 3.2.2. Surveys

Surveys will be administered to three participant groups:

*Health service CEO or nominee:* At the commencement of the study (T1) and following the first year of implementation (T3), CEOs or their nominee will be asked to complete two surveys:

1. The *Program Sustainability Assessment Tool* (PSAT) [54] assesses the sustainability of public health programmes through 40 Likert-scale questions across eight domains: (1) environmental support, (2) funding stability, (3) partnerships, (4) organisational capacity, (5) programme evaluation, (6) programme adaptation, (7) communications, and (8) strategic planning. The PSAT has been applied at both the community and state level [55,56].
2. The *Expectations Regarding Aging Questionnaire* (ERA-12) [57] is a 12-item survey that measures expectations regarding ageing. It includes three 4-item scales (expectations regarding physical health, mental health, and cognitive function) and one global expectation-regarding-ageing scale combining all 12 items. The scale has been used in several studies and is recommended for use in a systematic review of global scales of ageism [58].

The surveys will be available online using the Australian-hosted REDcap platform. Paper copies will be provided where requested.

*Primary health clinicians survey*: At the commencement of the study (T1) and after the first year of implementation (T3), primary health clinicians will be asked to complete three surveys:

3. A custom-designed pre- and post-Indigo 4Ms implementation survey based on action items in the comprehensive care standard for primary care [59] and previous research into the effectiveness of a comprehensive care plan in a Victorian acute hospital [60].
4. The *Assessment of Interprofessional Team Collaboration Scale II* (AITCS-II) [61]. AITCS-II measures collaboration in health care team practice through 23 questions across three subscales: (1) partnership, (2) cooperation, and (3) coordination. The tool has been used in the clinical and primary care settings [62,63].
5. The *Expectations Regarding Aging Questionnaire* (ERA-12) [57].

*Older persons*: Eligible older people will receive a survey from their primary care team comprising questions 1–7 of the Australian Hospital Patient Experience Question Set (AHPEQS) [64]. Hospitals and health care services use the AHPEQS to obtain feedback from patients about their experiences of treatment and care [65,66]. The question set has been translated into 20 languages and is available in easy English, large print, and braille. The survey will be provided in a format suitable for the older person.

#### 3.2.3. Interviews

*Health service executives (HSEs)*: Each of the five health services’ CEOs or their nominees will attend a monthly Project Control Group established by the project lead agency to provide governance and strategic leadership. Each meeting has an agenda item for a discussion led by the Chief Investigator. This discussion will be included in the Terms of Reference for the PCG, which will be agreed upon at the commencement of the study. As part of the consent process, participants will be advised that the discussion forms part of the research process and will be audio-recorded. The discussion guide is structured through the adapted Program Sustainability Assessment Tool.

*Implementation team (IT)*: After the first year of implementation (T3), the implementation team at each health service will participate in semi-structured small-group interviews. The number of participants will vary depending on the number employed at each service and their availability. A review of the monitoring data will inform the discussion guide.

*Primary health clinicians (PHCs)*: After the first year of implementation (T3), the primary health teams at each health service will be interviewed in small groups. The number of participants will vary depending on the number employed at each service and their availability. The discussion guide is structured using the Assessment of Interprofessional Team Collaboration Scale. An analysis of relevant survey data will inform its use.

*Older people*: Semi-structured, individual interviews will be undertaken with older people who have completed a survey and agreed to be contacted by a researcher for an interview. The discussion guide is structured through the concepts of the Indigo 4Ms Tool and questions 1–7 of the Australian Hospital Patient Experience Question Set.

*Facilitators (Fs)*: Semi-structured interviews will be completed after the first year of implementation (T3).

#### 3.2.4. Monitoring Tools

Two monitoring tools will be used to capture continuous data on the implementation process: a coding sheet and field notes.

*Coding sheet (CS)*: Throughout the implementation period, facilitators at each site will be asked to complete a monitoring tool based on domains from the Framework for Reporting Adaptations and Modifications-Enhanced (FRAME) [67] and the Framework for Reporting Adaptations and Modifications to Evidence-based Implementation Strategies (FRAME-IS) [68]. The tool will collect data about the content and process of adaptions and modifications (the who, what, where, when, and how) and the decision-making processes (the why) as the Indigo 4Ms Tool is implemented in their health services. Each facilitator will be encouraged to complete the survey fortnightly or as they make decisions and take action.

*Field notes (FNs)*: Researchers will record their observations in writing as they participate in PCG meetings and the facilitators’ monthly community of practice meetings.

Table 2 summarises the data collection methods at each time point of the research, mapped against the PRISM/RE-AIM framework.

### 3.3. Data Analysis

To begin with, data will be analysed on an individual case basis. The survey data will be analysed using MS Excel (v2405) spreadsheets. Responses will inform the areas for further exploration in interviews. Each interview will be recorded, transcribed verbatim, and entered into NVivo (v1.7(1533)). In NVivo, data will be analysed using thematic analysis based on the survey constructs and study objectives. Field notes and ongoing reflection within the research team will support the dependability and credibility of the analytical process.

Data from each case will then be synthesised and analysed across the collective cases. This meta-synthesis will be undertaken using Donabedian’s theory (1988) of relationships between structure (context and resources), process (comprehensive care planning), and outcomes (improved multidisciplinary teamwork and older person’s positive experience) to describe the core components of an effective implementation of the Indigo 4Ms Tool for rural primary health care.

## 4. Expected Results

To address Aim 1 (evaluation of the effectiveness of the Indigo 4Ms Tool), data will be analysed to identify the organisational contexts and resources that support the implementation of the Indigo 4Ms Tool, the reach of the tool to primary health clinicians, any changes to comprehensive care planning for older people within the participating services, any changes to collaboration between and within multidisciplinary teams, and whether use of the tool improves staff satisfaction and scope of practice. The collected data are expected to inform knowledge of whether the tool enhances the experience of working in partnership with older people on their care planning.

To address Aim 2 (evaluation of the implementation strategies), data will be analysed to identify the uptake of the Indigo 4Ms Tool outside of primary health teams; describe the extent to which the use of the tool is maintained or embedded in health service policy and practice; understand which strategies, under what circumstances, best supported the adoption of the Indigo 4Ms Tool; describe which adaptions to the tool and its implementation strategies were necessary to support adoption and sustainability; and how these adaptions improved effectiveness.

The expected outcomes of this evaluation are as follows:

1. A documented evidence-based process that can be replicated in other rural and remote settings to improve the capacity of the multidisciplinary workforce to deliver integrated care for older people that addresses functional ability.
2. To generate a sustainable, cost-effective approach to incorporating a focus on functional ability in routine care for rural community-dwelling older people.

Understanding how to better support the functional ability of older people who live rurally through integrated, multidisciplinary care is critical in meeting the health and well-being needs of an increasing global cohort. This research will contribute new, geographically diverse understandings of how 4Ms approaches to caring for older people can be successfully implemented in primary care settings.

## Figures and Tables

**Table 1:** Implementation settings.

Health Service Identifier	Primary Health Implementation Setting
Service 1	Small communities receiving home care packages. Services include case management, nutrition, continence management, mobility and leisure activities, and clinical care.
Service 2	Primary health services in small town providing complex care, diabetes education, health promotion, occupational therapy, physiotherapy, and podiatry
Service 3	Primary health services to rural and remote areas include General Practice, physiotherapy, occupational therapy, fitness centre, community transport, diabetes education, personal home care, and counselling.
Service 4	Home and community care services delivered in regional centre including continence, district nursing, dietitian, occupational therapy, physiotherapy, podiatry, social work, and speech therapy.
Service 5	District Nursing Unit which provides care in rural communities across diverse geographies and townships.

**Table 2:** RE-AIM/PRISM evaluation objectives, measures, and data collection.

		Time	T1			T2	T3			T4		
	Objectives	Measures	Audit	Srvy	Itv	Monitor	Audit	Srvy	Itv	Audit	Srvy	Itv
*P*	1. Organisation contexts and resources	Readiness and feasibility	A1	HSE	HSE	HSE		HSE	HSE			
		Resources, barriers/enablers	A1	HSE	HSE	HSE		HSE	HSE			
R	2. Reach of the tool	No. (%) and discipline of clinicians who use I4Ms					A2			A2		
E	3. Changes to CCP	% CCPs with evidence of I4Ms	A2							A2		
		Evidence of person-centred care	A2							A2		
	4. Collaboration between and within MDT	Team interactions		PHC				PHC	IT PHC			
		Referrals to other disciplines and agencies	A2				A2		IT PHC	A2		
	5. Staff satisfaction	Staff satisfaction							PHC			
		Scope of practice							PHC			
	6. Partnership with older person in CCP	Older person’s satisfaction				OP		OP	OP			
A	7. Uptake of the tool	No. (%) and discipline of clinicians who use I4Ms outside study setting					A2		PHC			HSE
		Uptake by other areas of health service							PHC			HSE
I	8. Strategies and context for adoption	Adaptions of the I4Ms and implementation strategies				HSE F CS FN			IT PHC F			HSE
	9. Changes to tool and strategies											
M	10. Maintenance in policy and practice	I4Ms as routine practice								A2		HSE
		I4Ms in policy for CCP								A2		HSE

## Data Availability

No new data were created or analysed for this article. Data sharing does not apply to this article.
